# Next-generation freshwater bioassessment: eDNA metabarcoding with a conserved metazoan primer reveals species-rich and reservoir-specific communities

**DOI:** 10.1098/rsos.160635

**Published:** 2016-11-30

**Authors:** Nicholas K. M. Lim, Ywee Chieh Tay, Amrita Srivathsan, Jonathan W. T. Tan, Jeffrey T. B. Kwik, Bilgenur Baloğlu, Rudolf Meier, Darren C. J. Yeo

**Affiliations:** 1 Department of Biological Sciences, National University of Singapore, Singapore; 2 Lee Kong Chian Natural History Museum, National University of Singapore, Singapore

**Keywords:** eDNA, freshwater bioassessment, metabarcoding

## Abstract

Freshwater habitats are of high conservation value and provide a wide range of ecosystem services. Effective management requires regular monitoring. However, conventional methods based on direct observation or specimen collection are so invasive, expensive and labour-intensive that frequent monitoring is uncommon. Here, we test whether the evaluation of environmental DNA (eDNA) from water based on a simple protocol can be used for assessing biodiversity. We use universal metazoan primers for characterizing water eDNA across horizontal and vertical spatial dimensions in two reservoirs with known species diversity for two key taxa. eDNA obtained directly from 42 samples × 15 ml water (total = 630 ml) per reservoir yielded DNA signatures for more than 500 metazoan species, of which 105 could be identified to species/genus based on DNA barcodes. We show that eDNA can be used to assign each water sample to its reservoir of origin, and that eDNA outperforms conventional survey methods in single-sample richness comparisons, while revealing evidence for hundreds of unknown species that are undetected by conventional bioassessment methods. eDNA also confirms the presence of a recently discovered invasive snail species and provides evidence for the continued survival of a rare native species of goby not sighted in that habitat since 2007. eDNA thus promises to be a useful addition to the bioassessment toolbox for freshwater systems.

## 1. Introduction

Only a relatively small proportion of the Earth's surface is fresh water, but freshwater habitats are home to a disproportionately large number of species [1] while being extensively used by humans for a wide range of ecosystem services. It is thus important to regularly assess their ecological health and develop effective monitoring techniques. Central to effective biomonitoring, conservation and management is the ability to assess aquatic faunal communities regularly, with minimal impact, and at acceptable cost. Conventional methods relying on directly collecting target species or sampling communities are time-consuming, labour-intensive, invasive and rarely yield species-level information for invertebrates.

Advances in high-throughput DNA sequencing technologies now allow for the evaluation of environmental DNA (eDNA) either via metagenomics [2,3] or metabarcoding [4–8]. Both techniques rely on detecting species based on trace DNA in the environment. This can be particularly useful for biomonitoring, because obtaining eDNA from fresh water is cost-effective and minimally invasive. Use of freshwater eDNA for the detection of particular taxa (e.g. fishes, amphibians, insects [7,9–13]) is now routine, but it is unclear whether whole communities can be characterized using universal primers that amplify across Metazoa and are applied to eDNA obtained from water without filtration [9,10,14–17]. In addition, very little is known about the utility of eDNA in the tropics where higher temperatures are likely to expedite DNA degradation. Existing studies in the tropics have been largely taxon-specific and/or used filtration prior to DNA extraction [13,18]. Lastly, it remains to be seen whether eDNA samples from different freshwater bodies, sampling sites and sampling depths contain signatures which allow them to be distinguished from one another.

Here, we present a proof-of-concept for eDNA metabarcoding in tropical fresh waters with a single metazoan COI primer pair [14,15]. Our study focused on two urban freshwater reservoirs in Singapore where ongoing biodiversity studies have yielded species lists for validation of eDNA results. Based on historical physico-chemical data, one has somewhat better water quality than the other, but they do not represent extremes across Singapore's reservoirs [19]. In our study, we estimate the metazoan species richness in each reservoir based on multiple sites (seven per reservoir) and depths (two per site) and validate eDNA results for key taxa with species lists from multi-year comprehensive surveys. Lastly, we compare community structure between and within reservoirs and examine whether presence–absence data reveals habitat-specific signatures independently of sequencing read counts.

## 2. Material and methods

### 2.1. Water sampling

Water samples were collected from seven sites each in Bedok (1.3413° N, 103.9245° E) and Pandan (1.3154° N, 103.7434° E) Reservoirs, Singapore, with permission from PUB, Singapore's National Water Agency (permit number PUB/RP11-32). Six of the sites corresponded to littoral sites where electrofishing was performed by the Freshwater and Invasion Biology Laboratory (National University of Singapore), while the seventh site was close to the centre of the reservoir with the greatest depth (electronic supplementary material, table S1 and figure S1*a*,*b*: electronic supplementary materials and methods). Water samples were collected using Van Dorn horizontal water samplers; one sampler was used for surface samples (approx. 0.5 m beneath water surface) and another sampler was used for benthic samples. Prior to sampling, both samplers were rinsed twice with reverse osmosis (RO) water and the water from the second rinse was retained as negative controls. Water from each collection was dispensed into a clean 50 ml tube, then poured into three separate 50 ml tubes (containing 33 ml of absolute ethanol and 1.5 ml of 3 M sodium acetate) and filled to the mark [10]. Samplers were rinsed with RO water between each collection. Tubes were capped, inverted gently to mix and kept on ice until arrival at the laboratory, where they were stored at −80°C until DNA extraction.

### 2.2. DNA extraction and metabarcoding PCR

Samples were thawed completely then centrifuged at 5500*g* for 35 min at 4°C to pellet cells and DNA, and the supernatant was discarded. To minimize contamination risk, we used only fresh bottles of reagents, and performed all subsequent steps in a class II biological safety cabinet, with all inner surfaces decontaminated with 10% bleach solution, followed by 70% denatured ethanol and UV irradiation for 30 min. Samples from different reservoirs were processed on different days.

To each tube, including an additional empty one (extraction negative control), we added 900 µl CTAB buffer (0.1 M Tris pH 8; 1.4 M NaCl; 0.02 M EDTA; 20 g l^−1^ CTAB) to resuspend the pellet and transferred the mixture into a microcentrifuge tube, to which we added 20 µl 1 mg ml^−1^ proteinase K buffer (Invitrogen). Tubes were shaken to mix and incubated at 55°C for 3 h, shaking occasionally. Next, we added 600 µl of 25 : 24 : 1 phenol : chloroform : isoamyl alcohol (PCI; Biozol) to each tube. Contents of each tube were mixed and centrifuged for 10 min at 17 900*g*. A further 600 µl of PCI was added to the aqueous fraction, mixed and centrifuged again for 10 min at 17 900*g*. Absolute ethanol was then added to the aqueous fraction, to the 1.5 ml mark. Tubes were then stored at −30°C overnight to precipitate DNA.

Tubes were then centrifuged at 17 900*g* for 35 min to pellet DNA. Pellets were washed by adding 700 µl 70% ethanol, mixing and centrifugation at 17 900*g* for 10 min. Pellets were air-dried, re-suspended in nuclease-free water and stored at −30°C until PCR.

For amplification of the 313 bp COI barcode fragment, we used the degenerate metazoan primers mICOIintF: 5′-GGWACWGGWTGAACWGTWTAYCCYCC [14] and jgHCO2198: 5′-TAIACYTCIGGRTGICCRAARAAYCA [15]. Five replicate PCRs were performed for each DNA extract, sampling negative control and PCR negative control (without DNA extract), and individual PCRs used primers tagged at their 5′ ends with unique 9 bp identifier sequences. PCR mixtures comprised 1X reaction buffer (Bioer), 0.2 mM each dNTPs (Bioer), 0.2 µg µl^−1^ BSA (HyClone), 0.4 mM of each tagged primer (Integrated DNA Technologies), 1 U BioReady rTaq DNA Polymerase (Bioer) and 2 µl DNA extract, topped up to a final volume of 25 µl. Cycling conditions were as follows: 3 min at 95°C; 35 cycles of 1 min at 95°C, 1 min at 45°C, 1 min at 72°C; 3 min at 72°C. Amplification success was verified on agarose gel by the presence of a 365 bp band. DNA extracts for which PCR was difficult, such as those from benthic samples containing sediment, required protocol adjustments including reducing annealing temperature to 42°C, using up to 0.8 µg µl^−1^ BSA, up to 1% Tween-20 (Sigma-Aldrich), or up to 100-fold dilution of DNA extracts for enhanced relief of PCR inhibition.

### 2.3. High-throughput sequencing

An equal amount of each PCR product (*n* = 230 per reservoir) was pooled into a single multiplexed sample (one such sample per reservoir), immediately purified using SureClean Plus (Bioline) according to the manufacturer's protocol, then stored at −20°C until sequencing. PCR products were stored in separate freezer compartments from DNA extracts to minimize the risk of cross-contamination. One sequencing library per multiplexed sample was prepared using a TruSeq Nano DNA Library Preparation Kit (Illumina) according to the manufacturer's protocol except that only six enrichment PCR cycles were performed. Sequencing was performed on a HiSeq 2500 system (Illumina) using the HiSeq Rapid SBS Kit v2 in Rapid Run Mode, to obtain paired 250 bp reads.

### 2.4. Preliminary data processing

OBITools v. 1.2.0 [20] was used to merge paired-end reads, demultiplex data based on primer tags and prune PCR and sequencing errors (electronic supplementary materials and methods). A preliminary BLAST search [21] was performed against a curated collection of 313 bp COI barcodes retrieved from GenBank [22] and local databases (Meier *et al*. 2014, unpublished data: Y. Yi [fishes]; D. Yeo [odonates]; B. Baloğlu [chironomids]), using BLASTN as implemented in BLAST +2.2.30 [23]. Sequences with best matches to non-metazoan phyla, or without matches at more than or equal to 80% identity to a metazoan database entry were discarded. The remaining sequences were aligned using MAFFT v7 with the FFT-NS-2 algorithm [24] and translated using MEGA 6 [25]. Poorly aligned sequences or those untranslatable using either the vertebrate or the invertebrate mitochondrial codes were discarded. To further minimize retention of erroneous sequences or chimaeras, we only used sequences obtained in at least three of the five PCR replicates. Retained sequences from both libraries were combined into a single dataset and clustered at a 3% uncorrected pairwise distance (*p*-distance) threshold [26] using objective clustering (Srivathsan, unpublished software; implementation of objective clustering described in Meier *et al*. [27]) to delimit sequences into putative species units, or molecular operational taxonomic units (MOTUs). We also examined the stability of MOTU counts at 2 and 4% *p*-distance thresholds to assess if estimates at 3% are reliable.

### 2.5. Identification of molecular operational taxonomic units and comparison with conventional surveys

To identify MOTUs, we performed BLAST searches against GenBank and local COI databases built from barcoded species occurring in Singapore. We only assigned the barcode identification when the sequence overlap was at least 310 bp and the identity was more than or equal to 97%. All remaining MOTUs were subjected to a search in the BOLD identification engine [28] with the same match percentage cut-off. The remaining MOTUs have sequence matches of 90–97% to metazoan barcodes were identified to higher taxa using the Statistical Assignment Package (SAP) 1.9.0 [29]. We configured SAP to download 50 homologues at more than or equal to 0.8 identity, and accepted only the phylum-level assignment at more than or equal to 0.95 posterior probability.

Comprehensive species lists based on years of study exist for fish and nuisance chironomid midges of the two reservoirs. We first compared the lists of species detected using eDNA with these lists. Afterwards, we compared the eDNA lists with those detected using conventional survey techniques applied over 1 day (electrofishing: Kwik JTB, Lim RBH, Liew JH, Kwang YYW, Ng WQ, Chen ML, Yeo DCJ, 2015, unpublished data; emergence trap collecting: Baloğlu *et al*. 2015, unpublished data). The comparisons are based on area under the curve analyses of presence–absence data with reference to species lists compiled from comprehensive reservoir survey data (electronic supplementary materials and methods).

### 2.6. Statistical analyses

To evaluate sampling completeness, MOTU accumulation curves were generated using the specaccum function as implemented in the vegan 2.3–4 package [30] in R v. 3.2.4 [31]. For MOTU community comparisons involving all MOTUs, and only common MOTUs (detected in at least four of seven surface or benthic samples), we generated two-dimensional non-metric multidimensional scaling (NMDS) plots based on Jaccard (for presence–absence data) and Morisita-Horn (for sequencing read count data) dissimilarities, using metaMDS as implemented in vegan. We computed Spearman's rank correlation coefficient (*ρ*) to correlate sequencing read counts with abundance and biomass estimates. To obtain non-parametric incidence-based richness estimates, we computed Chao2 [32,33] and ICE [34] using EstimateS 9.1 [35].

To further investigate whether samples carried reservoir-specific signatures, we first generated reservoir-specific reference profiles by assigning values of ‘0’ and ‘1’ to all MOTUs which were absent from, and present in, each reservoir, respectively. Next, we generated sample-specific profiles by repeating the process for each sample, then computed Jaccard dissimilarities between each sample-specific profile and reservoir-specific profile using vegdist as implemented in vegan. We also repeated this analysis using reservoir-specific reference profiles constructed using only common MOTUs.

## 3. Results

### 3.1. Sequencing data and molecular operational taxonomic unit delimitation

**Figure 1.**
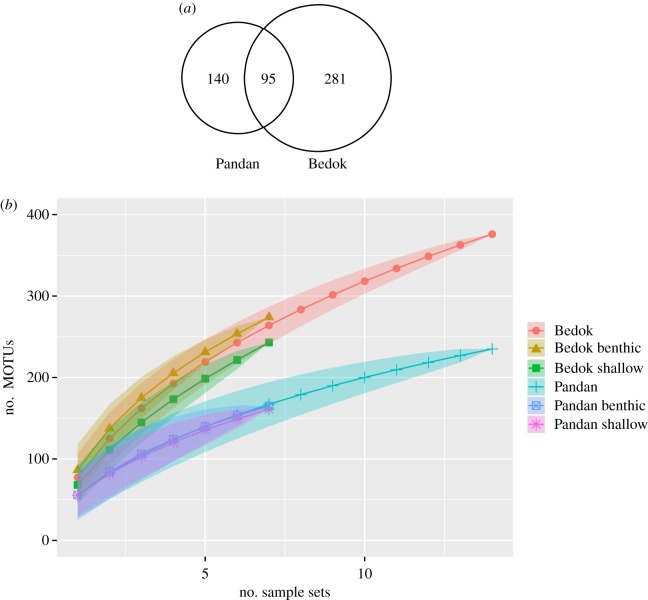
MOTU composition and richness comparisons between Bedok and Pandan Reservoirs. (*a*) Venn diagram displaying the number of MOTUs unique to each reservoir, and common to both. (*b*) MOTU accumulation curves show higher overall richness in Bedok when compared with Pandan. Both curves appear distant from the asymptote suggesting more MOTUs could surface with greater sampling effort. Depth-specific curves show significant similarity of the MOTU richness profiles to that of the combined sample set for that reservoir.

A total of 32 103 043 paired-end reads were generated from the COI amplicons obtained from water samples from both reservoirs. After filtering, 3 925 189 reads corresponding to 2133 unique sequences were retained, none of which corresponded to negative controls. At a 3% *p*-distance threshold, the unique sequences clustered into 516 MOTUs across both reservoirs (electronic supplementary material, file S1). At 2 and 4%, 546 and 501 MOTUs were obtained; thus suggesting that a 3% threshold is unlikely to have substantially inflated or deflated richness estimates. Overall, Bedok was found to be 60% more species-rich than Pandan (376 versus 235 MOTUs). Ninety-five of the MOTUs were common to both reservoirs, while the majority were reservoir-specific (figure 1*a*). MOTU accumulation curves suggest that further sampling would reveal additional MOTUs; they also show that MOTU accumulation from surface and benthic samples alone do not differ significantly from the combined accumulation (figure 1*b*). This is corroborated by the substantially higher Chao2 and ICE richness estimates compared to raw MOTU richness (electronic supplementary material, figure S2).

### 3.2. Comparison between eDNA detection and conventional surveys

**Figure 2.**
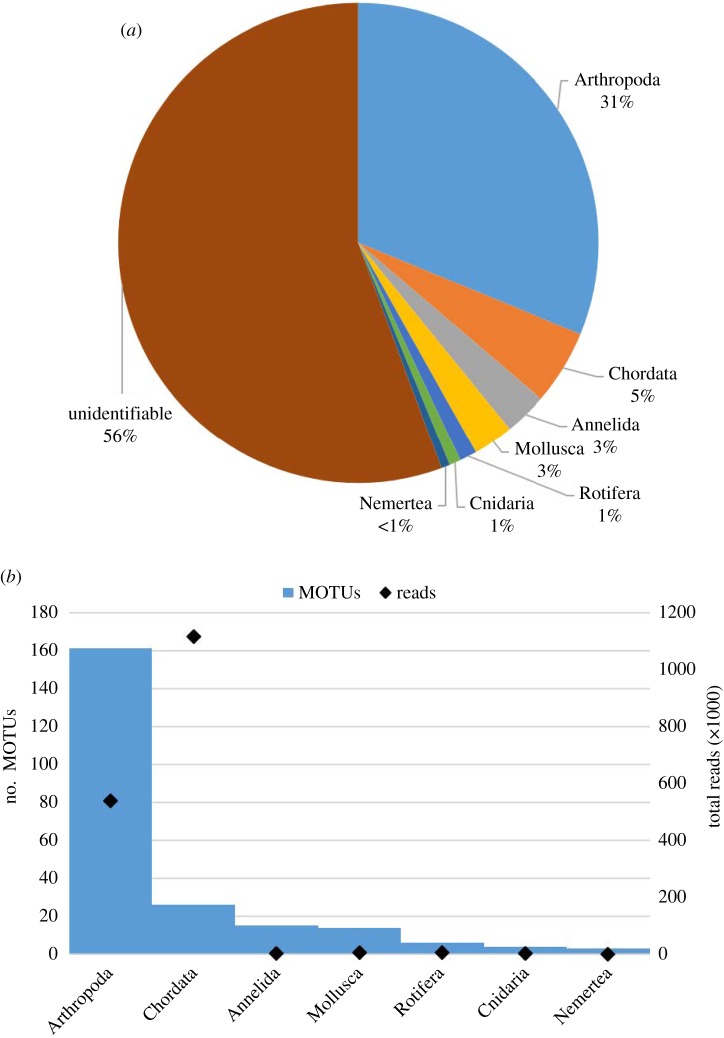
(*a*) Composition of MOTUs broken down to phylum level. Over half of the MOTUs remain unidentified, while arthropods and chordates have the largest and second largest representation of the seven represented by the identified MOTUs. (*b*) Total number of identified MOTUs from each phylum compared to the total number of sequencing reads corresponding to those MOTUs.

To compare species compositions based on eDNA and conventional surveys, we first assigned MOTUs to various taxonomic hierarchies. We could assign 229 MOTUs (44.4%) to a phylum, of which only 105 were identified to species (electronic supplementary material, file S1). In agreement with overall richness comparisons, we found Bedok to be more species-rich than Pandan in specific taxa such as fishes (20 versus 9 MOTUs), odonates (35 versus 8 MOTUs), chironomids (17 versus 11 MOTUs) and culicids (10 versus 0 MOTUs). We assigned 125 MOTUs their most probable identities using local unpublished databases, 79 using BOLD and the remaining 25 using GenBank. Over half of the identified MOTUs corresponded to arthropods, while chordates formed the second largest group (figure 2*a*). The largest number of sequencing reads per MOTU was observed for chordates (figure 2*b*).

When compared to comprehensive lists of known species constructed from surveys and historical records of Bedok and Pandan, 1 day of eDNA sampling at seven sites and two depths detected 19 of 27 (70%) fish species in Bedok, and 9 of 16 (56%) in Pandan. In comparison, electrofishing on the same day detected 33 and 62% of species in Bedok and Pandan, respectively. The same eDNA samples detected 12 of 27 (44%), and 8 of 18 (44%) of chironomid species in Bedok and Pandan, respectively, while 1 day's worth of emergence traps collected 37 and 17% of species from the comprehensive lists. Site-by-site comparisons further show that eDNA samples almost always outperformed electrofishing (table 1), and frequently outperformed single emergence traps (table 2) at the same site.

**Table 1.** Overall and site-by-site comparison between eDNA and electrofishing conducted on the same days, for the detection of fishes in Bedok and Pandan. Numerical values represent the number of species detected, as a fraction of the number of species known from comprehensive surveys of the two reservoirs (Bedok *n* = 27, Pandan *n* = 16; electronic supplementary material, file S2).

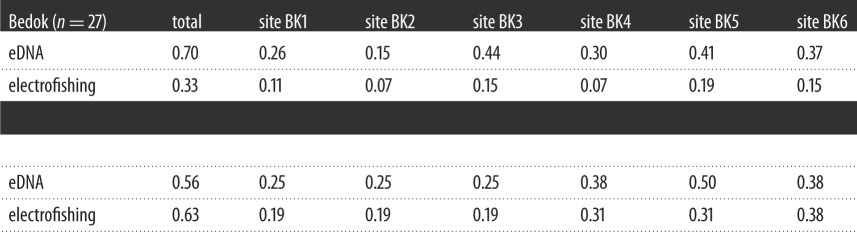

**Table 2.** Overall and site-by-site comparison between eDNA and emergence trapping for the detection of non-biting midges (Diptera: Chironomidae) in Bedok and Pandan. Numerical values represent the number of species detected, as a fraction of the number of species known from comprehensive surveys of the two reservoirs (Bedok *n* = 27, Pandan *n* = 18; electronic supplementary material, file S2). Since trapping was not conducted at the same time as eDNA sampling, we compared eDNA data to past-year trapping data from the nearest calendar days. Bedok emergence trap data were from 11 September 2013; Pandan emergence trap data were from 20 June 2013 (I) and 24 June 2014 (II).

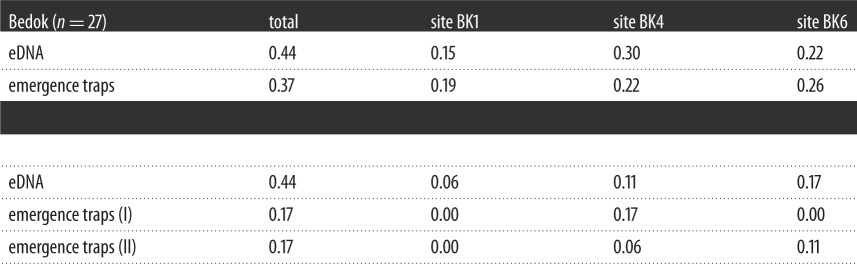

### 3.3. Community comparisons across space

**Figure 3.**
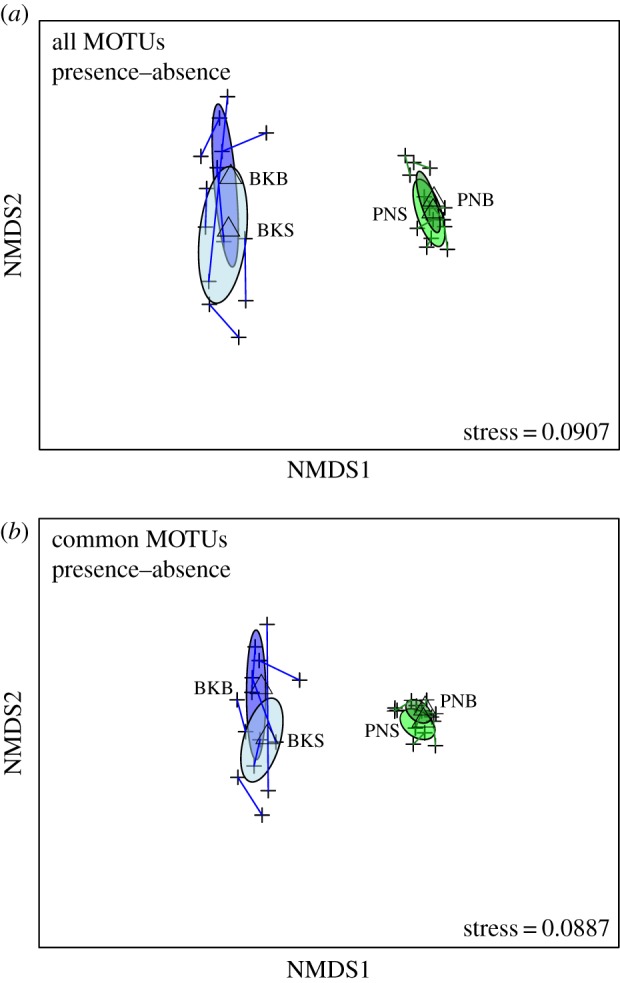
Two-dimensional NMDS plots constructed based on Jaccard dissimilarities between MOTU presence–absence profiles compare community structure between and within Pandan and Bedok Reservoirs. Crosses represent the centroids of MOTU communities at each sampling point, while lines connect surface and benthic sampling depths at the same site. Ellipses represent the sample set at each depth at each reservoir and triangles represent their centroids (PNS, Pandan surface; PNB, Pandan benthic; BKS, Bedok surface; BKB, Bedok benthic). Comparisons were made using (*a*) all MOTUs and (*b*) only MOTUs occurring in at least four of seven surface or benthic samples.

**Figure 4.**
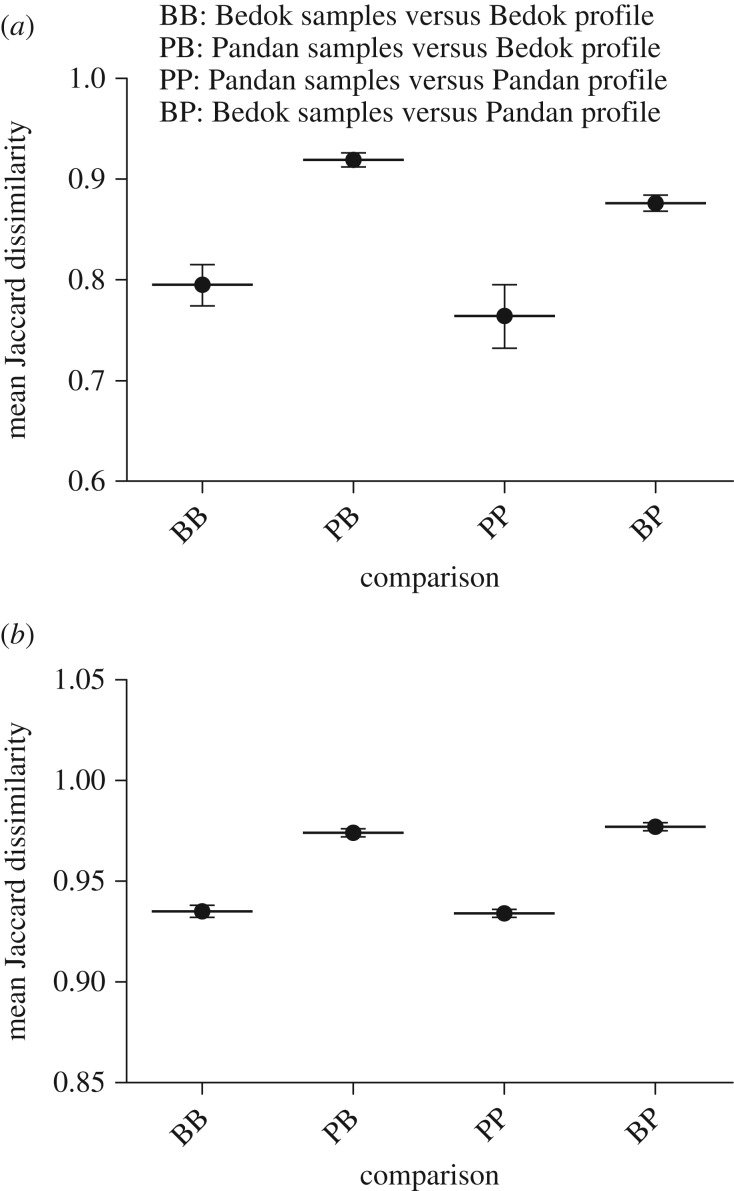
(*a*) Mean Jaccard dissimilarities reveal that, as expected, Bedok samples are significantly more similar to the Bedok reservoir-specific profile than Pandan samples (*p* < 0.0001), and Pandan samples are significantly more similar to the Pandan reservoir-specific profile than Bedok samples (*p* < 0.0001). (*b*) The same is observed when only common MOTUs are used to construct reservoir-specific profiles (Pandan *p* < 0.0001; Bedok *p* < 0.0001). Error bars represent s.e.m.

When comparing all MOTUs using the presence–absence data, the NMDS plot reveals distinct separation between Bedok and Pandan MOTU communities (figure 3*a*). When considering only common MOTUs, similar patterns are observed (figure 3*b*). The use of rank sequencing read counts led to the same qualitative conclusions (electronic supplementary material, figure S3*a*,*b*), which are also supported by the lower Renkonen similarity (*p*) between reservoirs (all MOTUs *p* = 0.177; common MOTUs *p* = 0.177) than that between nearly any two sampling points within a reservoir (all MOTUs electronic supplementary material, table S2; common MOTUs electronic supplementary material, table S3). Based on Jaccard dissimilarities computed from the presence–absence data, all Bedok samples are more similar to the Bedok reservoir-specific profile than Pandan samples, and likewise all Pandan samples are more similar to the Pandan reservoir-specific profile (figure 4*a*; electronic supplementary material, file S2). Excluding rare MOTUs from comparison yielded a qualitatively similar result (figure 4*b*). Together with the patterns revealed by the NMDS plots, this is compelling evidence that eDNA samples carry reservoir-specific signatures based on species compositions alone.

## 4. Discussion

With just 630 ml of water collected from each reservoir in a virtually non-invasive manner within 6 h, eDNA metabarcoding detected over 500 metazoan MOTUs using a single primer pair. One in five of these MOTUs can be identified with confidence to species across seven phyla (figure 2*a*,*b*). Analyses of the amplified products using high-throughput sequencing led to diversity inferences that are in agreement with our current knowledge of the two reservoirs. Bedok Reservoir is known to be more species-rich based on species lists obtained with conventional methods for several taxa including fish and chironomid species. We also demonstrate that eDNA provides more complete species lists per sampling event than conventional survey methods. This, coupled with the species-level resolution provided by DNA barcodes suggests that the analysis of eDNA has the potential to become a powerful bioassessment tool for water quality.

**Figure 5.**
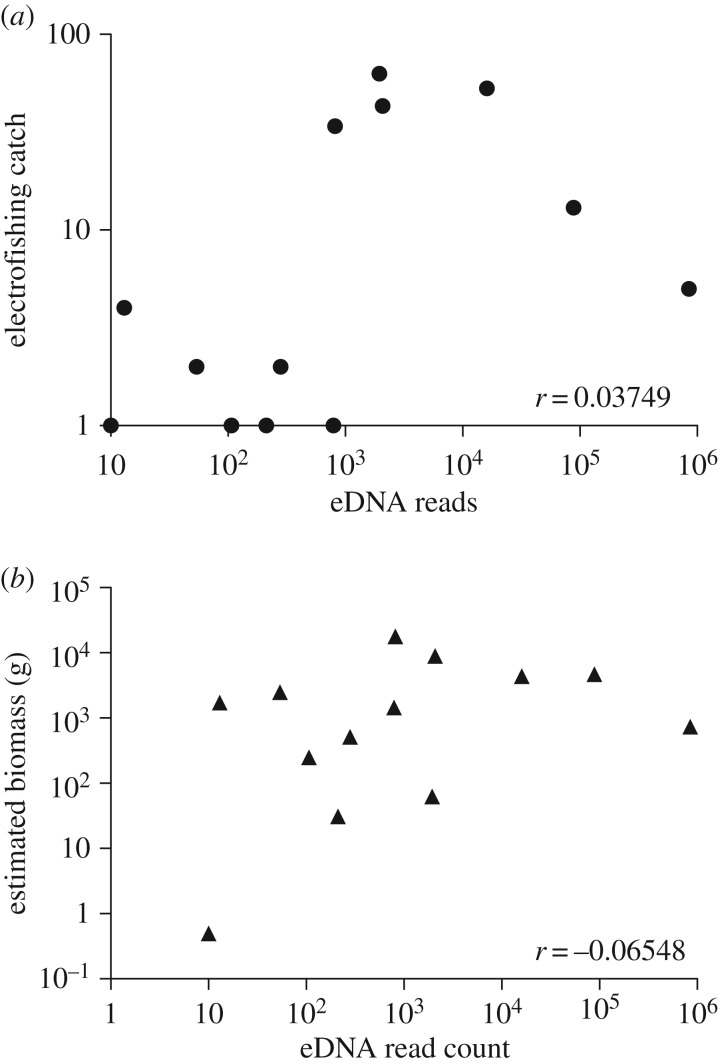
(*a*) Same-day electrofishing catch counts (as proxy for abundance) plotted against eDNA sequencing read counts for fishes reveal poor rank correlation. (*b*) Total catch weights (as proxy for biomass) plotted against read counts also reveal poor rank correlation. Together, these suggest that eDNA sequencing read counts are a poor proxy for both abundance and biomass estimated using a conventional fish survey method.

We found that MOTU presence–absence data reliably distinguishes eDNA samples from the two reservoirs based on species composition alone. Excluding rare MOTUs from comparisons led to the same conclusion but the reservoir-specific signatures were even stronger. This suggests that eDNA metabarcoding can reproducibly reveal habitat-specific signatures and allow a sample to be matched to its source without requiring the use of sequencing read counts as a proxy for abundance data. Conventional bioassessment techniques frequently rely on abundance information for taxa identified with low taxonomic resolution (e.g. genus, family) [36,37]. With eDNA, such abundance information can come in the form of overall MOTU richness or MOTU distribution information derived from incidence frequencies across sites; for example, some species are found across all sampling sites in a reservoir while others are restricted to one site. Another source of abundance information could be sequencing read counts, but we found, for instance, that sequencing read counts for fish MOTUs correlate poorly with electrofishing catch counts and weights (figure 5*a*,*b*). This result is not surprising given that quantitative biases in metabarcoding data are expected [38]. For example, eDNA capture and PCR have strong stochastic effects [39] which are confirmed by finding poor correlations between read counts from different PCR replicates for the same DNA extraction. Bias caused by PCR drift can, however, be partially mitigated by increasing the number of replicates [40]. We submit that future biomonitoring based on eDNA will more likely rely on MOTU richness and/or distribution because it takes advantage of a key strength of eDNA; i.e. detection of large numbers of species at high taxonomic resolution.

Our study reveals signatures for more than 500 MOTUs, and the stability of MOTU counts across *p*-distance thresholds attests to the reliability of the data. Biologists may question the value of using unidentified MOTUs. However, even unidentified MOTUs can be matched across different samples and habitats and can be used for the analysis of community structure. Currently, only about one in five MOTUs could be identified to species; over half of these based on local barcode databases populated using specimens collected in Singapore. Identifying MOTUs to species is desirable because additional information on the species can be obtained from the scientific literature which often helps with improving the value of a MOTU for bioassessment. It is thus desirable to develop better barcode databases [41]. eDNA is also useful for finding rare and unexpected species. For example, we detected evidence for the presence of the bigmouth stream goby (*Pseudogobiopsis oligactis*) in Bedok Reservoir. This rare, native species has not been recorded in Bedok Reservoir since 2007 and was thought to be extirpated in Singapore until its rediscovery in a stream elsewhere [42,43]. We also detected in Pandan Reservoir the serrated crownsnail (*Pyrgophorus platyrachis*), a newly recorded invasive species [44]. Thus, eDNA is useful for monitoring invasive species, and detecting the presence of rare species that may be missed in conventional surveys. Additionally, the large number of unidentified MOTUs highlights the need to study zooplankton such as rotifers and microcrustaceans. We predict that as the use of eDNA gains prominence, freshwater zooplankton may become more important for freshwater bioassessment, which may be desirable because they are likely to respond quickly to changes in water quality.

Our study is not without problems; our MOTU list contained a small number of unexpected identifications (*n* = 15; 2.9% of all MOTUs), including highly environmentally sensitive odonates which are not known to be found in the reservoirs (D. Yeo and R. W. J. Ngiam 2016, personal communication). This highlights one potential problem with eDNA when assessing interconnected freshwater habitats such as reservoirs and catchments. The persistence of eDNA in water [45] can generate false positives. These are difficult to eliminate without sophisticated theoretical models and comprehensive information on water movement. The persistence of eDNA is likely to also explain the high diversity and surprisingly large species overlap found between surface and benthic samples collected from hypoxic or anoxic waters. Lastly, even with many control measures in place, eDNA samples are likely to get cross-contaminated during laboratory procedures (PCR, library preparation, sequencing) although we only found a small number of suspect sequences that generated a low amount of stochastic noise.

Our study demonstrates the viability of using metabarcoded eDNA for characterizing whole metazoan communities in lentic habitats—an important first step towards incorporating such molecular methods in biotic indices for environmental-related decision-making. At this point, however, further research is needed in order to understand the temporal and spatial stability of eDNA signals within and across multiple reservoirs. Such information will be essential for establishing standardized field protocols and for developing eDNA-based bioassessment tools. Traditionally, bioassessment is based on indices of biological integrity that compare species communities of reference sites (usually with low anthropogenic impact) with communities of assessed (impacted) sites, or that directly score assessed sites based on the presence or the absence (and sometimes abundance) of broad taxonomic groups with varying levels of environmental sensitivity/tolerance. The community information is currently obtained via time- and resource-consuming field surveys that concentrate on macroinvertebrates. They are collected, sorted and identified often only to high taxonomic levels (e.g. order, family and genus) using traditional morphological methods. It is here that eDNA can potentially help. Collecting water samples is fast and eDNA provides information on more taxa at greater taxonomic resolution (i.e. down to species level). eDNA thus holds the promise for high-resolution bioassessment at lower manpower cost. One obstacle would be the need for developing new eDNA-based indices comparing reference and impacted sites and calibrating environmental responses at higher resolution (i.e. species level). However, new indices may not be immediately needed because biological identifications at greater taxonomic resolution (eDNA: species-level) can be translated to higher taxonomic level (traditional surveys: order, genus and family). This implies that the currently used indices of biological integrity can be used as long as eDNA provides information on the same taxonomic groups and provides suitable abundance information. This should be assessed in follow-up studies that compare survey results based on traditional and eDNA techniques for the same polluted and unpolluted habitats.

## 5. Conclusion

We show that freshwater eDNA metabarcoding can provide a snapshot of metazoan biodiversity including bioindicator, rare, invasive and nuisance species. It also allows for comparing species richness and correlating it with water quality. The species communities detected using eDNA reveal habitat-specific signatures without relying on abundance estimates, implying that eDNA-derived signals are highly reproducible. Our study implies that eDNA metabarcoding holds much promise as a minimally invasive method for ecological surveys and/or frequent biomonitoring.

## Supplementary Material

Supplementary_Figures

## Supplementary Material

Supplementary_File_1

## Supplementary Material

Supplementary_File_2

## Supplementary Material

Supplementary_Materials_and_Methods

## Supplementary Material

Supplementary_Tables

## Data accessibility

Data from this study have been deposited on the Dryad Digital Repository: http://dx.doi.org/10.5061/dryad.40hs5 [46].
