# Supplementary material for: Next-generation freshwater bioassessment: eDNA metabarcoding with a conserved metazoan primer reveals species-rich and reservoir-specific communities
Source: R Soc Open Sci. 2016 Nov 30;3(11):160635. doi: 10.1098/rsos.160635 (PMC5180151; doi:10.1098/rsos.160635)
Supplement: Supplementary_Figures [file rsos160635supp1.pdf]

Figure S2: Raw MOTU richness compared to asymptotic non-parametric richness estimates, Chao2 and ICE.

Figure S3: Two-dimensional NMDS plots constructed based on Morisita-Horn dissimilarities community structure between and within Pandan and Bedok Reservoirs based on the sequencing read counts representing each MOTU. Crosses represent the centroids of MOTU communities at each sampling point, while lines connect surface and benthic sampling depths at the same site. Ellipses represent the sample set at each depth at each reservoir and triangles represent their centroids, where PNS: Pandan surface; PNB: Pandan benthic; BKS: Bedok surface; BKB: Bedok benthic. Comparisons were made using **(a)** all MOTUs, and **(b)** only common MOTUs.

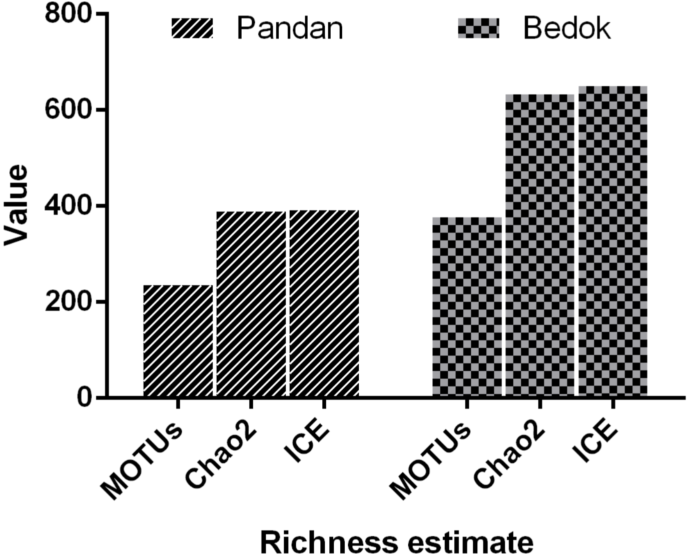
800
600
400
200
0
Value
Pandan
Bedok
MOTUs
Chao2
ICE
MOTUs
Chao2
ICE
Richness estimate

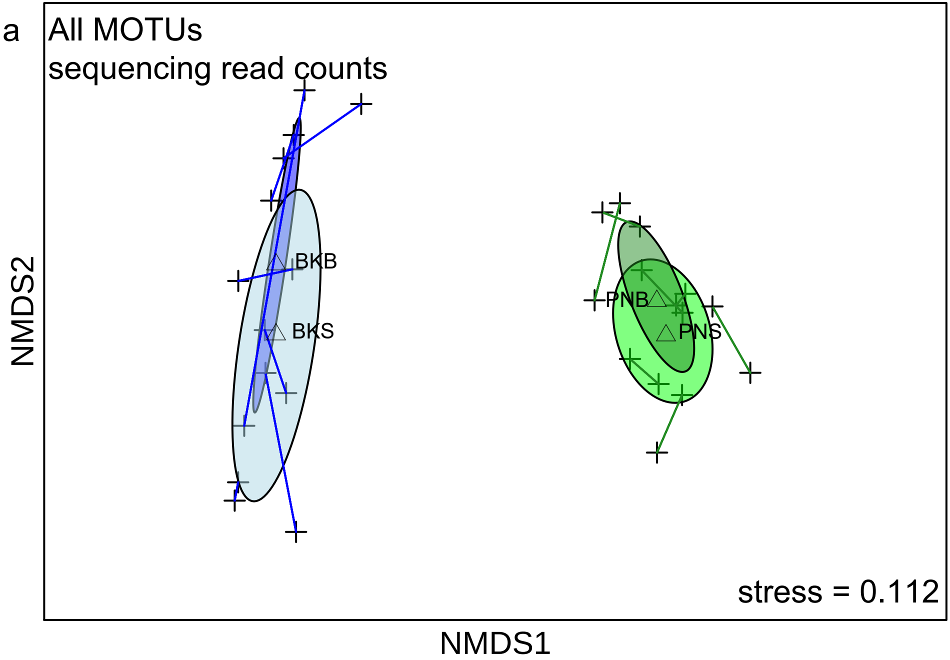
a
All MOTUs
sequencing read counts
BKB
BKS
PNB
PNS
stress = 0.112
NMDS2
NMDS1

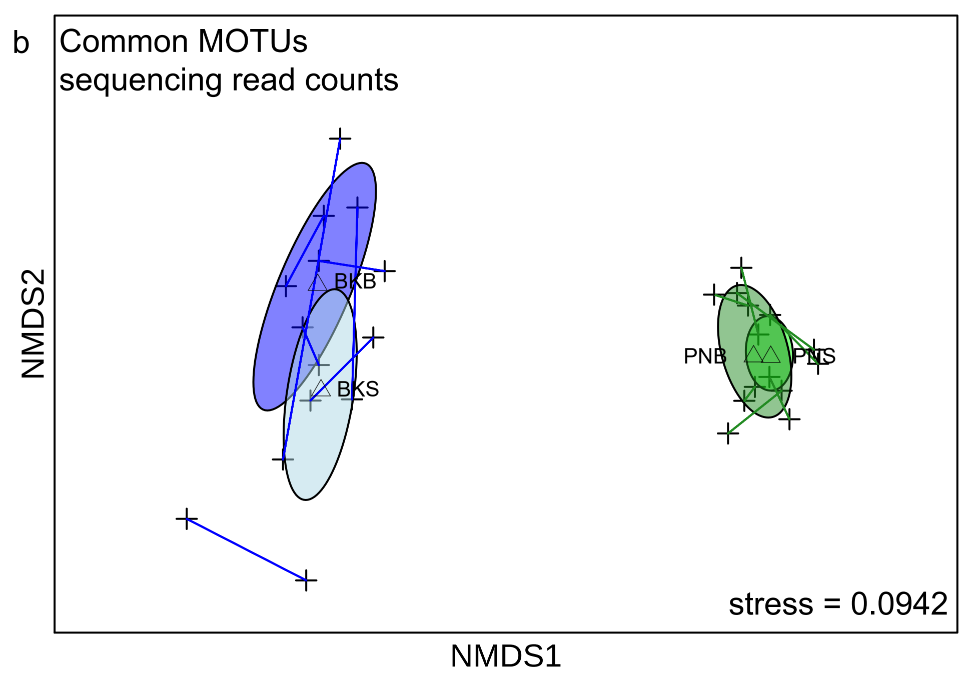
b
Common MOTUs
sequencing read counts
BKB
BKS
PNB
PNS
stress = 0.0942
NMDS1
NMDS2
