# Supplementary material for: Next-generation freshwater bioassessment: eDNA metabarcoding with a conserved metazoan primer reveals species-rich and reservoir-specific communities
Source: R Soc Open Sci. 2016 Nov 30;3(11):160635. doi: 10.1098/rsos.160635 (PMC5180151; doi:10.1098/rsos.160635)
Supplement: Supplementary_Materials_and_Methods [file rsos160635supp4.docx]

**Supplementary Materials and Methods for:**

Lim NKM, Tay YC, Srivathsan A, Tan JWT, Kwik JTB, Baloğlu B, Meier R, Yeo DCJ (2016). Next-generation freshwater bioassessment: eDNA metabarcoding with a conserved metazoan primer reveals high species richness and reservoir-specific communities. *R. Soc. Open Sci.*

**Use of conventional survey data for comparison**

Reservoir fish and chironomid species lists

Reservoir fish lists were obtained from a recent comprehensive survey incorporating Pandan and Bedok Reservoirs conducted in 2013 [Kwik et al., unpublished]. Lists of chironomid species were obtained from a recent comprehensive survey conducted between 2012 and 2014, also involving both reservoirs [Baloglu, unpublished].

Electrofishing data

We obtained electrofishing data from each site, which as mentioned correspond to eDNA sampling sites. We compared eDNA species lists with electrofishing catch lists from the same days. For comparisons with abundance and biomass, we took catch counts as a proxy for abundance and total catch weight as a proxy for biomass, since mark-recapture experiments failed to generate reliable population estimates.

Chironomid survey data

As no conventional chironomid surveys were performed in June and September 2015 to coincide with eDNA sampling, we compared eDNA data in Bedok with a single set of traps (sampling performed on 11^th^ September 2013), and in Pandan with two sets of traps (sampling performed on 20^th^ June 2013 and 24^th^ June 2014). We chose trapping data from the same, or the closest, calendar days to eDNA sampling site for the most appropriate comparison, since seasonality is observed in the chironomid composition in the reservoirs [Baloglu, pers. comm.]. In addition, we excluded the centre site in Bedok Reservoir from comparisons due to a mass emergence event in 2013 which was likely to substantially skew data.

**Table S1**: List of water sampling locations at Pandan and Bedok Reservoirs, including dates of sampling, approximate depths at which benthic samples were collected, and the precise GPS coordinates of each sampling location.

| *Reservoir* | *Sampling site ID* | *Sampling date* | *Approx. benthic sampling depth (m)* | *GPS coordinates* |
| --- | --- | --- | --- | --- |
| Pandan | PN1 | 22 June 2015 | 2.5 | N01º18.722', E103º44.148' |
| Pandan | PN2 | 22 June 2015 | 1.5 | N01º18.227', E103º44.672' |
| Pandan | PN3 | 22 June 2015 | 2.5 | N01º18.604', E103º44.755' |
| Pandan | PN4 | 24 June 2015 | 2.0 | N01º18.963', E103º44.150' |
| Pandan | PN5 | 24 June 2015 | 2.0 | N01º19.172', E103º44.362' |
| Pandan | PN6 | 24 June 2015 | 2.0 | N01º19.088', E103º44.755' |
| Pandan | PN7 | 24 June 2015 | 10.0 | N01º18.699', E103º44.614' |
| Bedok | BK1 | 14 Sep 2015 | 1.0 | N01º20.440', E103º55.265' |
| Bedok | BK2 | 14 Sep 2015 | 4.0 | N01º20.370', E103º55.532' |
| Bedok | BK3 | 14 Sep 2015 | 2.0 | N01º20.333', E103º55.837' |
| Bedok | BK4 | 14 Sep 2015 | 3.0 | N01º20.512', E103º55.842' |
| Bedok | BK5 | 14 Sep 2015 | 2.0 | N01º20.698', E103º55.622' |
| Bedok | BK6 | 14 Sep 2015 | 2.0 | N01º20.736', E103º55.326' |
| Bedok | BK7 | 14 Sep 2015 | 17.0 | N01º20.531', E103º55.600' |

**a**


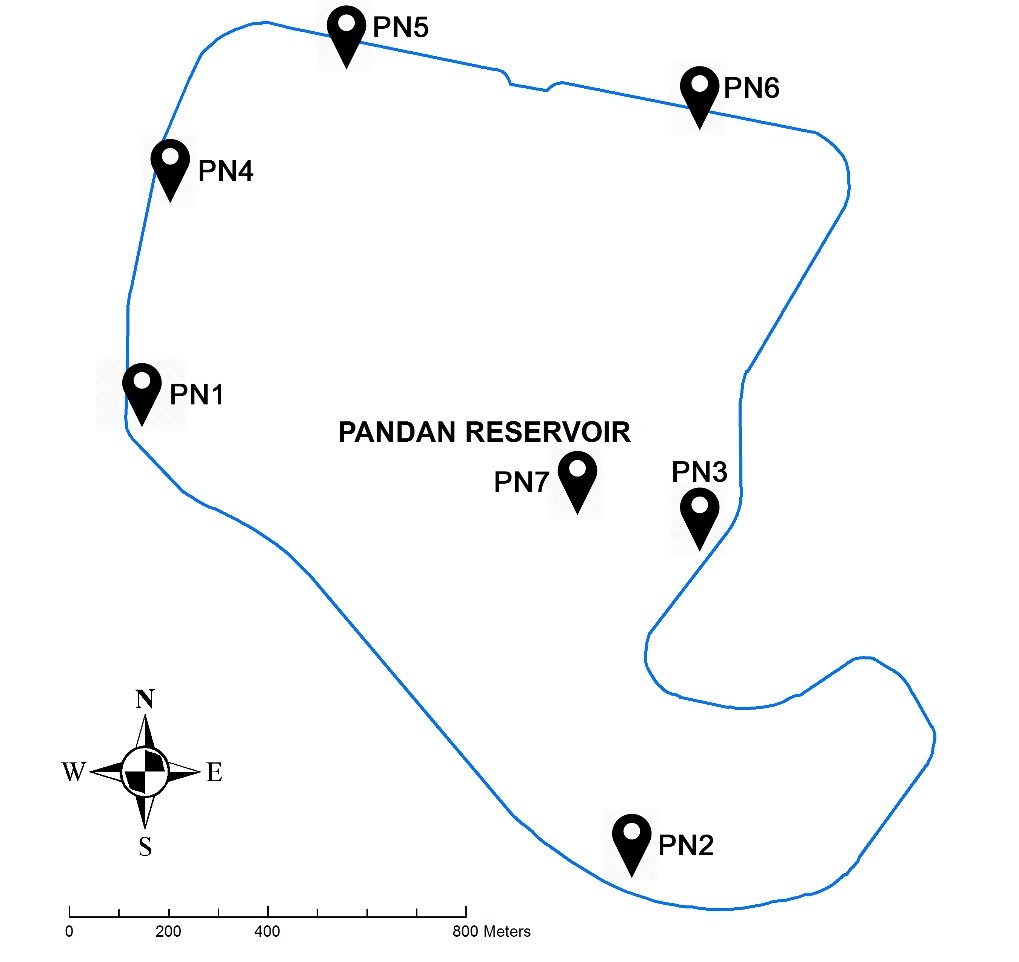


**b**


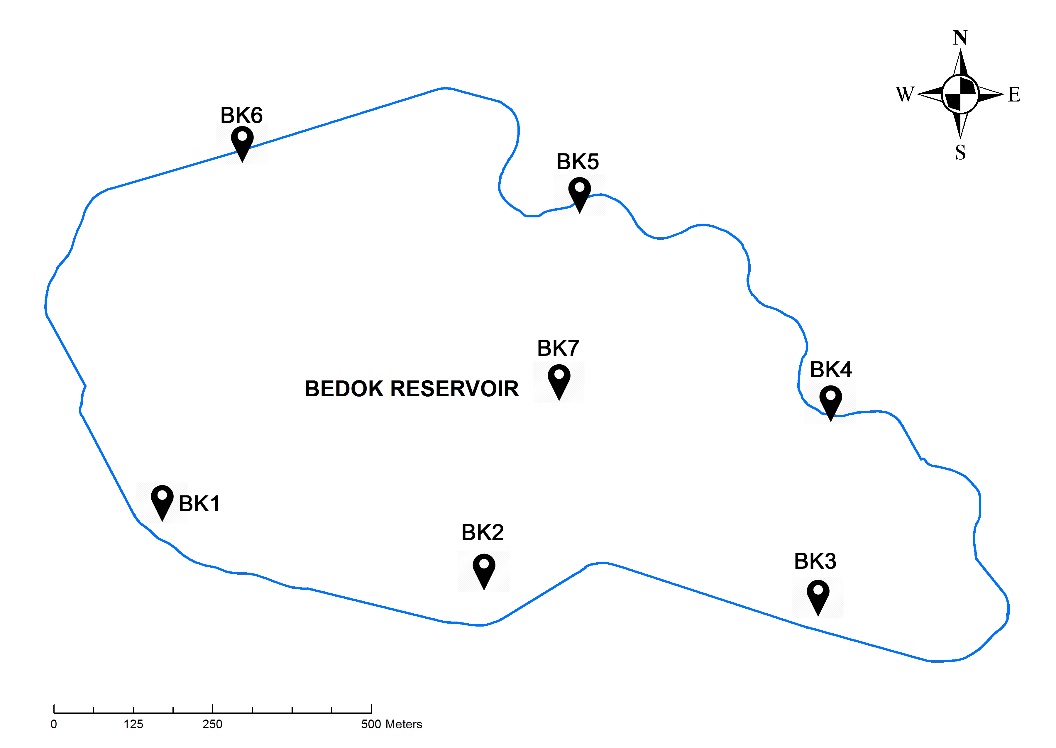


**Figure S1:** Maps of sampling sites in **(a)** Pandan (1.3154° N, 103.7434° E) and **(b)** Bedok (1.3413° N, 103.9245° E) Reservoirs.

**List of OBITools 1.2.0 commands used in metabarcoding analysis**

OBITools commands were used in this order. This example was performed for the Pandan Reservoir sequencing library.

illuminapairedend –-score-min=40 -r PN_reverse.fastq PN_forward.fastq > PN.fq

For merging forward and reverse reads. Performed once for each sequencing library.

obigrep -p ‘mode!=”joined”’ PN.fq > PN_ali.fq

For discarding sequences that are unaligned and simply concatenated, and retaining only properly aligned paired-end reads.

ngsfilter -t SI_Pandan_obitools-ngsfilter.txt -u unidentified.fq PN_ali.fq > PN_ali_assigned.fq

Assigns sequences to their samples of origin based on primer and assigned tag sequences.

obiuniq -m sample PN_ali_assigned.fq > PN_ali_assigned_uniq.fa

Counts duplicate sequences and de-replicates them, retaining sample information and counts in FASTA headers.

obiannotate -k count -k merged_sample PN_ali_assigned_uniq.fa > $$ ; mv $$ PN_ali_assigned_uniq.fa

Retains only sample ID and sequence counts in FASTA headers.

obigrep -l 300 -p ‘count>=10’ PN_ali_assigned_uniq.fa > PN_ali_assigned_uniq_10_300.fa

Retains only sequences 300 bp or longer and with counts of at least 10.

obiclean -s merged_sample -r 0.05 -H PN_ali_assigned_uniq_10_300.fa > PN_ali_assigned_uniq_10_300_clean.fa

Variant calling step, assigning “head”, “singleton” or “internal” status to each sequence. “Head” sequences are those with no variants with counts greater than 5% of their own count.

obiannotate -k obiclean_count -k obiclean_status PN_ali_assigned_uniq_10_300_clean.fa > $$ ; mv $$ PN_ali_assigned_uniq_10_300_clean.fa

Cleans the FASTA headers, keeping only sample IDs with corresponding sequence counts, and head/internal/singleton status.

obitab -o PN_ali_assigned_uniq_10_300_clean.fa > PN_ali_assigned_uniq_10_300_clean.tab

Converts the cleaned FASTA file into a tabular format for subsequent splitting by sample, removal of “internal” sequences, and downstream analysis.
