# Supplementary material for: Next-generation freshwater bioassessment: eDNA metabarcoding with a conserved metazoan primer reveals species-rich and reservoir-specific communities
Source: R Soc Open Sci. 2016 Nov 30;3(11):160635. doi: 10.1098/rsos.160635 (PMC5180151; doi:10.1098/rsos.160635)
Supplement: Supplementary_Tables [file rsos160635supp5.docx]

**Supplementary Tables for:**

Lim NKM, Tay YC, Srivathsan A, Tan JWT, Kwik JTB, Baloğlu B, Meier R, Yeo DCJ (2016). Next-generation freshwater bioassessment: eDNA metabarcoding with a conserved metazoan primer reveals high species richness and reservoir-specific communities. *R. Soc. Open Sci.*

**Table S2:** Renkonen similarity matrices comparing communities (all MOTUs) between sampling points within Bedok (BK) and Pandan (PN) Reservoirs, including between sites (1–7), and between depths (B: benthic, S: surface). Numerical values of 1.00 indicate perfect community similarity.

|  | BK1B | BK1S | BK2B | BK2S | BK3B | BK3S | BK4B | BK4S | BK5B | BK5S | BK6B | BK6S | BK7B | BK7S |
| --- | --- | --- | --- | --- | --- | --- | --- | --- | --- | --- | --- | --- | --- | --- |
| BK1B | 1.00 |  |  |  |  |  |  |  |  |  |  |  |  |  |
| BK1S | 0.44 | 1.00 |  |  |  |  |  |  |  |  |  |  |  |  |
| BK2B | 0.39 | 0.46 | 1.00 |  |  |  |  |  |  |  |  |  |  |  |
| BK2S | 0.39 | 0.50 | 0.42 | 1.00 |  |  |  |  |  |  |  |  |  |  |
| BK3B | 0.41 | 0.54 | 0.57 | 0.44 | 1.00 |  |  |  |  |  |  |  |  |  |
| BK3S | 0.68 | 0.62 | 0.42 | 0.47 | 0.48 | 1.00 |  |  |  |  |  |  |  |  |
| BK4B | 0.25 | 0.42 | 0.24 | 0.22 | 0.24 | 0.24 | 1.00 |  |  |  |  |  |  |  |
| BK4S | 0.58 | 0.65 | 0.43 | 0.48 | 0.52 | 0.85 | 0.20 | 1.00 |  |  |  |  |  |  |
| BK5B | 0.39 | 0.69 | 0.47 | 0.44 | 0.49 | 0.53 | 0.46 | 0.49 | 1.00 |  |  |  |  |  |
| BK5S | 0.40 | 0.58 | 0.37 | 0.44 | 0.47 | 0.67 | 0.22 | 0.70 | 0.54 | 1.00 |  |  |  |  |
| BK6B | 0.41 | 0.46 | 0.40 | 0.40 | 0.38 | 0.41 | 0.30 | 0.38 | 0.49 | 0.39 | 1.00 |  |  |  |
| BK6S | 0.40 | 0.58 | 0.37 | 0.44 | 0.44 | 0.63 | 0.23 | 0.67 | 0.53 | 0.72 | 0.45 | 1.00 |  |  |
| BK7B | 0.45 | 0.36 | 0.31 | 0.29 | 0.36 | 0.43 | 0.57 | 0.42 | 0.35 | 0.29 | 0.47 | 0.33 | 1.00 |  |
| BK7S | 0.66 | 0.63 | 0.42 | 0.46 | 0.47 | 0.87 | 0.25 | 0.80 | 0.54 | 0.61 | 0.48 | 0.64 | 0.49 | 1.00 |

|  | PN1B | PN1S | PN2B | PN2S | PN3B | PN3S | PN4B | PN4S | PN5B | PN5S | PN6B | PN6S | PN7B | PN7S |
| --- | --- | --- | --- | --- | --- | --- | --- | --- | --- | --- | --- | --- | --- | --- |
| PN1B | 1.00 |  |  |  |  |  |  |  |  |  |  |  |  |  |
| PN1S | 0.44 | 1.00 |  |  |  |  |  |  |  |  |  |  |  |  |
| PN2B | 0.65 | 0.20 | 1.00 |  |  |  |  |  |  |  |  |  |  |  |
| PN2S | 0.61 | 0.35 | 0.61 | 1.00 |  |  |  |  |  |  |  |  |  |  |
| PN3B | 0.57 | 0.41 | 0.64 | 0.61 | 1.00 |  |  |  |  |  |  |  |  |  |
| PN3S | 0.24 | 0.21 | 0.41 | 0.28 | 0.40 | 1.00 |  |  |  |  |  |  |  |  |
| PN4B | 0.46 | 0.48 | 0.22 | 0.30 | 0.22 | 0.11 | 1.00 |  |  |  |  |  |  |  |
| PN4S | 0.26 | 0.22 | 0.22 | 0.22 | 0.18 | 0.15 | 0.19 | 1.00 |  |  |  |  |  |  |
| PN5B | 0.21 | 0.21 | 0.19 | 0.26 | 0.24 | 0.15 | 0.16 | 0.86 | 1.00 |  |  |  |  |  |
| PN5S | 0.20 | 0.09 | 0.27 | 0.23 | 0.25 | 0.25 | 0.08 | 0.78 | 0.82 | 1.00 |  |  |  |  |
| PN6B | 0.47 | 0.28 | 0.41 | 0.43 | 0.43 | 0.19 | 0.29 | 0.68 | 0.66 | 0.58 | 1.00 |  |  |  |
| PN6S | 0.10 | 0.10 | 0.10 | 0.10 | 0.09 | 0.07 | 0.09 | 0.79 | 0.82 | 0.77 | 0.56 | 1.00 |  |  |
| PN7B | 0.53 | 0.40 | 0.27 | 0.29 | 0.27 | 0.13 | 0.55 | 0.47 | 0.47 | 0.40 | 0.63 | 0.40 | 1.00 |  |
| PN7S | 0.50 | 0.54 | 0.21 | 0.30 | 0.21 | 0.11 | 0.78 | 0.22 | 0.16 | 0.08 | 0.29 | 0.10 | 0.56 | 1.00 |

**Table S3:** Renkonen similarity matrices comparing communities (common MOTUs only) between sampling points within Bedok (BK) and Pandan (PN) Reservoirs, including between sites (1–7), and between depths (B: benthic, S: surface). Numerical values of 1.00 indicate perfect community similarity.

|  | BK1B | BK1S | BK2B | BK2S | BK3B | BK3S | BK4B | BK4S | BK5B | BK5S | BK6B | BK6S | BK7B | BK7S |
| --- | --- | --- | --- | --- | --- | --- | --- | --- | --- | --- | --- | --- | --- | --- |
| BK1B | 1.00 |  |  |  |  |  |  |  |  |  |  |  |  |  |
| BK1S | 0.45 | 1.00 |  |  |  |  |  |  |  |  |  |  |  |  |
| BK2B | 0.40 | 0.46 | 1.00 |  |  |  |  |  |  |  |  |  |  |  |
| BK2S | 0.40 | 0.54 | 0.42 | 1.00 |  |  |  |  |  |  |  |  |  |  |
| BK3B | 0.42 | 0.56 | 0.58 | 0.47 | 1.00 |  |  |  |  |  |  |  |  |  |
| BK3S | 0.70 | 0.64 | 0.42 | 0.50 | 0.49 | 1.00 |  |  |  |  |  |  |  |  |
| BK4B | 0.25 | 0.44 | 0.24 | 0.23 | 0.24 | 0.24 | 1.00 |  |  |  |  |  |  |  |
| BK4S | 0.60 | 0.68 | 0.43 | 0.51 | 0.54 | 0.88 | 0.20 | 1.00 |  |  |  |  |  |  |
| BK5B | 0.40 | 0.73 | 0.49 | 0.48 | 0.51 | 0.55 | 0.47 | 0.51 | 1.00 |  |  |  |  |  |
| BK5S | 0.41 | 0.60 | 0.37 | 0.47 | 0.49 | 0.70 | 0.21 | 0.73 | 0.55 | 1.00 |  |  |  |  |
| BK6B | 0.42 | 0.48 | 0.41 | 0.41 | 0.39 | 0.43 | 0.30 | 0.39 | 0.50 | 0.40 | 1.00 |  |  |  |
| BK6S | 0.41 | 0.60 | 0.38 | 0.48 | 0.47 | 0.66 | 0.23 | 0.70 | 0.56 | 0.76 | 0.46 | 1.00 |  |  |
| BK7B | 0.46 | 0.38 | 0.32 | 0.31 | 0.36 | 0.44 | 0.59 | 0.44 | 0.36 | 0.30 | 0.49 | 0.35 | 1.00 |  |
| BK7S | 0.68 | 0.66 | 0.43 | 0.50 | 0.50 | 0.90 | 0.26 | 0.84 | 0.56 | 0.64 | 0.49 | 0.67 | 0.51 | 1.00 |

|  | PN1B | PN1S | PN2B | PN2S | PN3B | PN3S | PN4B | PN4S | PN5B | PN5S | PN6B | PN6S | PN7B | PN7S |
| --- | --- | --- | --- | --- | --- | --- | --- | --- | --- | --- | --- | --- | --- | --- |
| PN1B | 1.00 |  |  |  |  |  |  |  |  |  |  |  |  |  |
| PN1S | 0.44 | 1.00 |  |  |  |  |  |  |  |  |  |  |  |  |
| PN2B | 0.68 | 0.22 | 1.00 |  |  |  |  |  |  |  |  |  |  |  |
| PN2S | 0.66 | 0.39 | 0.71 | 1.00 |  |  |  |  |  |  |  |  |  |  |
| PN3B | 0.59 | 0.42 | 0.66 | 0.67 | 1.00 |  |  |  |  |  |  |  |  |  |
| PN3S | 0.24 | 0.21 | 0.43 | 0.30 | 0.41 | 1.00 |  |  |  |  |  |  |  |  |
| PN4B | 0.47 | 0.48 | 0.24 | 0.31 | 0.22 | 0.11 | 1.00 |  |  |  |  |  |  |  |
| PN4S | 0.26 | 0.22 | 0.23 | 0.23 | 0.18 | 0.14 | 0.19 | 1.00 |  |  |  |  |  |  |
| PN5B | 0.21 | 0.21 | 0.19 | 0.26 | 0.24 | 0.15 | 0.16 | 0.86 | 1.00 |  |  |  |  |  |
| PN5S | 0.20 | 0.09 | 0.27 | 0.26 | 0.25 | 0.25 | 0.08 | 0.79 | 0.83 | 1.00 |  |  |  |  |
| PN6B | 0.48 | 0.28 | 0.42 | 0.44 | 0.43 | 0.19 | 0.29 | 0.70 | 0.68 | 0.60 | 1.00 |  |  |  |
| PN6S | 0.10 | 0.10 | 0.10 | 0.10 | 0.08 | 0.07 | 0.09 | 0.79 | 0.83 | 0.78 | 0.57 | 1.00 |  |  |
| PN7B | 0.54 | 0.41 | 0.28 | 0.30 | 0.27 | 0.13 | 0.56 | 0.48 | 0.48 | 0.41 | 0.64 | 0.41 | 1.00 |  |
| PN7S | 0.51 | 0.55 | 0.22 | 0.31 | 0.21 | 0.11 | 0.78 | 0.22 | 0.16 | 0.08 | 0.30 | 0.10 | 0.57 | 1.00 |
